# Linguistic, Content and Face Validity of the Swedish Version of a Quality-of-Life Assessment for Children, Teenagers and Adults with Spina Bifida

**DOI:** 10.3390/ijerph21050624

**Published:** 2024-05-15

**Authors:** Michaela Dellenmark-Blom, Marie Andersson, Konrad M. Szymanski, Charlotta Levén Andréasson, Magdalena Vu Minh Arnell, Sofia Sjöström, Kate Abrahamsson

**Affiliations:** 1 Department of Pediatrics, Institute of Clinical Sciences, University of Gothenburg, 416 85 Gothenburg, Sweden; marie.kristina.andersson@vgregion.se (M.A.); magdalena.vuminh-arnell@vgregion.se (M.V.M.A.); or sofia.sjostrom@vgregion.se (S.S.); or kate.abrahamsson@vgregion.se (K.A.); 2 Department of Pediatric Surgery, Sahlgrenska University Hospital, Queen Silvia Children’s Hospital, 416 85 Gothenburg, Sweden; charlotta.leven.andreasson@vgregion.se; 3 Division of Pediatric Urology, Riley Hospital for Children, Indiana University Health, Indianapolis, IN 46202, USA; szymanko@iu.edu

**Keywords:** spina bifida, quality of life, rare disease, neurogenic bladder, translation, validity, patient-reported outcome

## Abstract

Spina bifida includes a spectrum of different neural tube defects. Myelomeningocele is the most serious type and is associated with a risk of paralysis and sensory dysfunction below the affected level, bladder/bowel dysfunction, brain dysmorphology, and impaired health-related quality of life (HRQoL). The aim of this study was to describe the establishment of linguistic, content and face validity of the Swedish version of a Quality-of-Life Assessment for children (QUALAS-C, *n* = 10 items), teenagers (QUALAS-T, *n* = 10 items) and adults with spina bifida (QUALAS-A, *n* = 15 items) based on the original US English versions. The process included close collaboration with the original instrument developer and complied with international standards on patient-reported outcome measurements. The procedure includes forward translation, expert and patient/parent review and reconciliation, back translation, back translation review and cognitive debriefing interviews with 16 people with spina bifida aged 8 to 33, providing them with the possibility of evaluating the clarity, adequacy, and comprehensiveness of QUALAS-C, QUALAS-T and QUALAS-A, respectively. The interviews lasted a median of 15 min (range 8–16) for QUALAS-C, 10 min (range 9–15) for QUALAS-T and 24 min (range 9–38) for QUALAS-A. Four main issues/topics needed attention and discussion after both the forward and back translation. Following the back translation review, all issues were resolved. The patient feedback revealed recognition of the HRQoL issues included in QUALAS, and also difficulties in understanding some questions. After the patients’ evaluation, four items were reworded for clarity. No study participant reported a wish to add to or remove questions from QUALAS. Hence, the Swedish versions of QUALAS became conceptually equivalent to the original US English versions and achieved linguistic, content and face validity. While empowering the voices of people with spina bifida, these results also enable their HRQoL to be properly assessed in research and clinical care in Sweden and in international studies.

## 1. Introduction

Spina bifida is a neural tube defect, which originates from the failure of the neural tube to close completely during the first month of embryonic development [1]. It has an internationally pooled prevalence of 38.9 per 100,000 live births (95% CI = 35.8, 42.4), with the prevalence being lower in studies in which folic acid fortification was mandatory (33.9 per 100,000) as opposed to studies in which fortification was voluntary or nonexistent (48.4 per 100,000) [1]. Spina bifida refers to a spectrum of abnormalities which can range from mild to serious. Myelomeningocele (MMC) is the most serious type, with exposed neural tissue or meninges in a fluid-filled sac that protrudes at the level of the affected vertebrae. The latter type is associated with risk of paralysis and sensory dysfunction below the affected level, bladder and bowel dysfunction predisposing to urinary and fecal incontinence, and urinary tract infections as well as kidney damage and failure. Brain dysmorphology, including hydrocephalus and Chiari II malformation, may contribute to cognitive deficits, including intellectual functioning disability [2,3]. MMC can today be surgically closed through fetal or postnatal surgical repair [4,5,6]. Before 1960, few people with spina bifida survived infancy, whereas today these children have a high life expectancy, extending into adulthood in many higher income countries [7,8,9,10]. A main focus of research, as well as care and treatment, has now become patients’ long-term physical, mental and social outcomes [11,12]. 

Health-related quality of life (HRQoL) is a multidimensional concept defined as the patient’s perception of the effect of illness and treatment from physical, psychological, and social perspectives [13,14]. A main approach to the assessment of a patient group’s HRQoL is to use generic measurements applicable to people independent of their health status, and to compare the outcomes of spina bifida patients and general populations. Another measurement approach involves using condition-specific HRQoL questionnaires that reflect issues of relevance to people living with a specific disease/condition. This approach is usually more likely to provide clinically relevant information [13,15]. Even if HRQoL studies in children with spina bifida apply different inclusion criteria to their populations, use different questionnaires and are conducted in different countries, most studies show that they have reduced HRQOL levels compared to the general/healthy population [11,16,17,18,19,20,21]. Previous research including patients with spina bifida in Sweden has shown that the use of generic instruments such as SF-36 showed poor feasibility, as the questions were not adjusted for this patient group [22] and the subsequent scores could be misinterpreted. Interestingly, until 2016, SF-36 was most commonly used in patients with neurogenic bladder and bowel conditions, including those with spina bifida [23]. In these patients, the questionnaires utilized in the assessment of patient-reported outcomes were heterogeneous and sometimes non-validated [23]. In the light of methodological advances in the field in the past years, two condition-specific instruments for spina bifida have been reported [21,24,25,26,27], both of which were derived from patient input, in accordance with current recommendations on patient-reported outcome measurements (PROMs) [14,28]. However, the measurement model “QUAlity of Life Assessment in Spina bifida for Children” (QUALAS) [21,24,29] is unique in providing an age-adapted measurement approach to HRQoL in children, teenagers and adults, while also offering the opportunity to evaluate HRQoL issues related to bladder and bowels across all age groups. Using QUALAS, Szymanski et al. demonstrated that urinary incontinence in children with spina bifida may worsen with age, starting at 10 years of age and continuing during the teenage years, when it approaches levels of HRQoL impact reported by adults with spina bifida. Fecal incontinence correlated with lower HRQOL regardless of age [30]. 

There are well-established recommendations for the translation and cultural adaptation of patient-reported outcome measurements to enhance their cross-cultural applicability and use [31]. These are paramount for the international standardization and pooling of data on rare diseases [32]. The QUALAS questionnaires were developed in the US and have since been translated and evaluated in Japan, Korea and Brazil [21,24,26,27,29,33,34], but not yet in Sweden. The aim of this study was therefore to establish the Swedish version of QUALAS for children, teenagers and adults with spina bifida, providing them with linguistic, content and face validity, prior to commencing a larger field test.

## 2. Materials and Methods

### 2.1. Ethics

The study was approved by the Swedish Ethical Review Authority (2020-00219, Approval 24 April 2020). Oral and written study information was given to families of children with spina bifida and adults with spina bifida, and informed written consent was collected from the children’s parents, teenagers aged 15–17, and adults with spina bifida 18 years or older.

### 2.2. QUALAS

The condition-specific measurement model QUALAS was originally developed in US English. It encompasses three different age-specific self-reported assessments for patients with spina bifida, the QUALAS-C [21] for children (8–12 years old), QUALAS-T [24] for teenagers (13–17 years old), and QUALAS-A [29] for adults aged ≥ 18 years. Models QUALAS-C and QUALAS-T consist of 10 items divided into two domains and QUALAS-A of 15 items divided into three domains, as shown in Table 1. Permission was obtained from the instrument developer Dr. Konrad M Szymanski to translate the questionnaires from US English into Swedish and to validate according to a predefined study protocol.

### 2.3. Framework and Definitions

**Figure 1:**
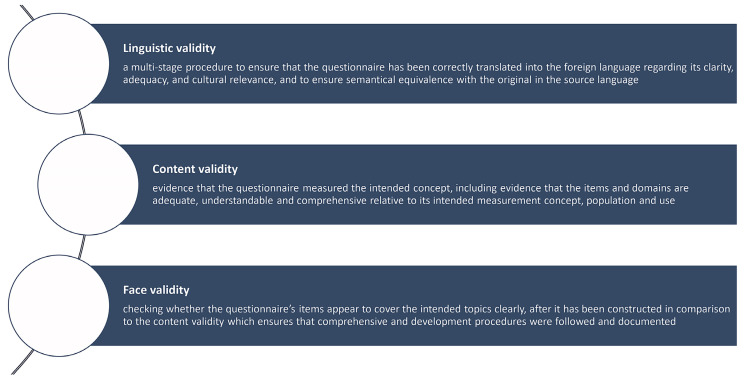
Definition of linguistic, content and face validity used as framework for the study.

The evaluation of the linguistic, content and face validity of QUALAS complies with international recommendations as to patient-reported outcome measurements [28,31]. Language translations of a PROM should provide conceptual and semantic equivalence with the original source language, referring to the absence of differences in meaning and content between the source language and the translated version [31]. To prove content validity, there should be evidence that the items and domains are adequate and comprehensive relative to their intended measurement concept (e.g., HRQoL), population (e.g., spina bifida) and use (e.g., interview-based approach) [28]. The definitions used for the establishment of the linguistic, content and face validity [35] of QUALAS are described in Figure 1.

### 2.4. Translation

All steps in the translation procedure of QUALAS were documented in a standardized format, using Microsoft Excel. 

#### 2.4.1. Step 1—Two Independent Forward Translations

Two senior urologists (Kate Abrahamsson, MD, Consultant in Pediatric Surgery/Urology, Professor of Pediatric Surgery; Marie Andersson, MD, PhD, Consultant in Pediatric Surgery/Urology, Fellow of the academy of pediatric urology), who were native speakers of Swedish (target language) and fluent in US English, independently translated the QUALAS-C, QUALAS-T and QUALAS-A from US English into Swedish while attempting to secure conceptual and linguistic equivalence with the original. 

#### 2.4.2. Step 2—Reconciliation

Using the two forward translations, a Swedish multidisciplinary project committee consisting of two senior pediatric urologists (Kate Abrahamsson, Marie Andersson), one senior urotherapist (Magdalena Vu Minh Arnell, RN, Senior Pediatric Nurse Specialist, Urotherapist PhD) one patient-reported outcome measurement methodologist (Michaela Dellenmark-Blom, RN, Senior Pediatric Nurse Specialist, PhD, Associate Professor) and one pediatric nurse (Lotta Andreasson, RN, Pediatric Nurse Specialist, MSc), reconciled these translations into one new Swedish language version. 

#### 2.4.3. Step 3—Patient/Parent Feedback and Consensual New Language Version 

The versions of QUALAS-C, QUALAS-T and QUALAS-A developed by the multidisciplinary committee were tested in individual interviews with representative people with spina bifida. Tests were led by one researcher (M.D.-B) for QUALAS C (two parents/one child), T (one teenager) and A (one adult). All study participants were native Swedish speakers and fluent in US English. They received the translated questionnaires in advance by post with instructions on their task and then gave their view of the Swedish translations during the interview. They were asked to give feedback on whether the questionnaire, including each item, was worded in a way they understood and in language they would use and if not, what their suggestions for improvements were. Following a review of the patient/parent testing, the multidisciplinary committee had new discussions, including input from and discussions with the original instrument developer where necessary. In this way, consensual new Swedish versions of QUALAS-C, QUALAS-T and QUALAS-A were defined.

#### 2.4.4. Step 4—Back Translation and Back Translation Review

The consensual Swedish versions of QUALAS-C, QUALAS-T and QUALAS-A were back-translated by a third professional translator, a native US English speaker fluent in Swedish, who had never seen the original US English versions. The back-translator had 30 years of experience translating Swedish–English in a wide variety of fields, including the medical field. The back-translation review of QUALAS was conducted by the local Swedish project committee and the original instrument developer to ensure conceptual and semantical equivalence between the original US English and Swedish versions of QUALAS-C, QUALAS-T and QUALAS-A. Therefore, in order to detect and correct any misunderstandings as to the translated questionnaires, specific issues in the back translations were highlighted and raised for discussion by the Swedish project committee and sent to the instrument developer. Following revisions, the Swedish versions of QUALAS-C, QUALAS-T and QUALAS-A were considered ready to be tested in cognitive debriefing interviews (Step 5). 

### 2.5. Cognitive Debriefing Interviews

#### 2.5.1. Step 5—Cognitive Debriefing Interviews

Cognitive debriefing is a qualitative and quantitative research technique used to provide insights into how respondents understand and answer questionnaires, including whether it corresponds to what the instrument developer intended [36]. Translations of a questionnaire into a new language [31] must optimize wording to ensure the respondent’s understanding of the questionnaire’s instructions, questions and response options [28,36]. The new consensual language versions of QUALAS-C, QUALAS-T and QUALAS-A were therefore tested by asking patients and parents about any difficulties in understanding the components of the questionnaires. This was done to improve the clarity of the Swedish version of QUALAS and to establish content and face validity. 

##### Setting and Study Participants 

There are seven university hospitals in Sweden, and at four of these sites, there are tertiary pediatric surgical centers for children. The study participants were recruited from the Queen Silvia Children’s Hospital, Sahlgrenska University Hospital in Gothenburg in the west of Sweden, which provides highly specialized medical and surgical care for children with spina bifida. In this study, the terms spina bifida and MMC are used as equivalents. All children, teenagers and adults who were recruited had taken part in a urotherapy follow-up program. Recruitment followed the study protocol defined by Dr. Szymanski, where the exclusion criteria for study participation were poor language ability in self-reports, developmental delay that interfered with answering questions, (open) surgery in the previous month, and a primary diagnosis other than spina bifida (primary tethered cord, sacral agenesis, medullary lipoma, anorectal malformation, spinal trauma or tumor). Furthermore, the study participants were selected to ensure representation of patients with spina bifida with and without ventriculoperitoneal shunts, and wheelchair users. Both male and female patients participated. The goal was to recruit at least five children 8–12 years old, five adolescents 13–17 years old and five adults with spina bifida. Out of nine eligible families of children with spina bifida, the first five randomly selected families of children aged 8–12 years were invited and accepted. There were 13 eligible teenagers aged 13–17, and nine of these were contacted and invited, six of whom agreed to participate. Among 10 eligible adults with spina bifida, eight were invited, and five accepted the invitation. The characteristics of the study participants are presented in Table 2. In the total sample, 50% were male, 75% had shunt-treated hydrocephalus, 75% needed a wheelchair and all of them used clean intermittent catheterization.

##### Data Collection

During 2022 and 2023, parents of children aged 8–17 with spina bifida and adults with spina bifida were contacted and invited to participate in a cognitive debriefing with a researcher (L.A., M.D.-B) who had not been part of their care and treatment. Following oral and written consent given by the children’s parents and by the adults with spina bifida, an interview was scheduled. To facilitate participation in the study, participants could choose to do the cognitive debriefing interview in conjunction with a visit to the outpatient clinic, online, or as a scheduled meeting with the researcher at a place of their choice. Children were permitted to choose whether they preferred to have their parent present during the interview or not. In cases where the parent was present, he/she was advised not to interfere with the child’s responses. The cognitive debriefing interviews were held as follows; QUALAS-C (one face-to-face interview at the hospital, four digital interviews, all with one parent present), QUALAS-T (one to face-to-face interview at the hospital, five digital interviews, all with one parent present) and QUALAS-A (one face-to-face, four digital interviews).

Two researchers performed the interviews separately, one of whom conducted fourteen interviews (L.A.) and one of whom held two interviews (M.D.-B). They applied a procedure which followed a predefined study protocol, including an introduction stating that the goal of the interview was to hear their opinion of QUALAS, and it was pointed out by the interviewer there were no right or wrong answers to the included questions. All participants were asked whether the Swedish translations of the instructions, response scale and items were easy to understand. They were also asked if they thought the domains contained questions which corresponded to their particular areas, and if any question was sensitive or uncomfortable to answer. During the interview, the researcher used cognitive probes that would elicit comments from the respondent’s perspective such as, ‘In your own words, how would you make them clearer or easier to understand?’ All interviews were sound recorded and field notes were taken, and the recorded comments from the study participants were transcribed by a research nurse (L.A.). The interviews lasted a median of 15 min (range 8–16) for QUALAS-C, 10 min (range 9–15) for QUALAS-T- and 24 min (range 9–38) for QUALAS-A.

##### Data Analysis

The number and proportion of respondents (*n*, %) in each age group who found the questionnaire instructions, questions, response options of the Swedish translation of QUALAS-C, QUALAS-T and QUALAS-A easy to understand, were analyzed. Similarly, descriptive statistics (*n*, %) were used to determine whether the participants perceived that the questions included in the domains corresponded with their names (yes/no), and if any question was sensitive/uncomfortable to answer (yes/no). Furthermore, the study participants’ open comments about the questions, if any, were analyzed by two researchers (M.D.-B, L.A.) using manifest content analysis, with their comments on content grouped according to shared features. These categories were then sorted into the “type of difficulty encountered” or “strength”, respectively. One respondent comment could only belong to one category. If the participants’ ratings (perceived clarity < 80% of the respondents) or open comments indicated a major problem with the translation, the need for revision was included in a careful discussion within the Swedish project committee and with the instrument developer.

### 2.6. Step 6—Production of the Final Swedish Language Version of QUALAS

The final version was created based on Steps 1 to 5. The suggestions were shared, and the results discussed, with the instrument developer. Together a consensus was established which resulted in the definitive Swedish language versions of QUALAS-C, QUALAS-T and QUALAS-A being included in the future larger-scaled field test. 

## 3. Results

### 3.1. Translation

The complete translation procedure for QUALAS-C, QUALAS-T and QUALAS-A is presented in detail in Supplemental Material S1. 

#### 3.1.1. Step 1—Two Independent forward Translations

In the forward translation of the QUALAS, there were overall few semantical differences between the two Swedish versions produced by the two translators, and none regarding the questionnaire instructions or response options. The semantic differences generally applied to the same expressions/equivalent questions in all age-specific versions of QUALAS, including semantically different translations in Swedish for “bother” (QUALAS-C; Q5, Q7; QUALAS-T; Q7, Q9, Q10; QUALAS-A, Q12, Q14, Q15), “health problem” (QUALAS-C, Q2; QUALAS-A, Q2) and “urine problems” (QUALAS-C, Q8; QUALAS-T, Q8; QUALAS-A, Q13), and in QUALAS-A, also “comfortable with your close friendships” (Q5). 

#### 3.1.2. Step 2—Reconciliation 

The semantic differences in translated questions led to careful discussion in the Swedish multidisciplinary project committee in order to reach a consensus on the most appropriate Swedish translations. The strategy to handle the semantic differences in the Swedish translation of “bother” (QUALAS-C, Q5, Q7; QUALAS-T; Q7, Q9, Q10; QUALAS-A, Q12, Q14, Q15) was to adjust the Swedish translation to its contextual use reflected in the specific questions, for “health problem” (QUALAS-C, Q2; QUALAS-A, Q2), to stay linguistically close to the US English translation, and for “urine problems” (QUALAS-C, Q8; QUALAS-T, Q8; QUALAS-A, Q13), to clarify the wording in Swedish by revealing its context to the bladder. Furthermore, “disposable underwear” is not used in Sweden, hence there was no good Swedish translation and it was judged necessary to remove this expression (QUALAS-C, Q6; QUALAS-T-Q6, QUALAS-A, Q11). For consistency, across all questions and age-specific versions, the appropriate tense of the Swedish questions was judged to be the “present perfect”. Additionally, some issues in the age-specific versions of QUALAS were discussed and resolved. 

In QUALAS-C, it was judged necessary by the Swedish multidisciplinary committee to simplify or change some expressions in the Swedish translations to make them more appropriate for children speaking Swedish, including the expressions for “catheterize” (Q3), “friends” (Q5), “urinary leakage” (Q7) and “stool” (Q10). The Swedish translation of “embarrassed” (Q1) referred to an emotive expression in Swedish, but the closest semantical Swedish translation compared to the original source language and was chosen. 

In QUALAS-T, two translations of “bother” were reviewed and adjusted to its context and semantical equivalence with the US English original source question (Q1, Q2). Similar to the QUALAS-C, some expressions in the Swedish translations needed to be adjusted to correspond to the language spoken among Swedish teenagers, including “friends” (Q2) and “urinary leakage” (Q8). Additionally, two translations of the questions within the domain Family and Independence (Q4, Q5), were difficult and needed adjustments for clarity (Supplementary File S1). 

In QUALAS-A, the best translation for “comfortable with your close friendships” (Q5) was selected, with the preference being a common word in the Swedish language. Also, questions using the expression “health problems” were discussed and an appropriate and consistent Swedish translation was found (Q2, Q7, Q10), see Supplementary Material S1). 

The translations of the questionnaire instructions and the response scales were easily merged into one Swedish version.

#### 3.1.3. Step 3—Patient/Parent Feedback and Consensus New Language Version 

Patient/Parent feedback on the Swedish translations of QUALAS was considered by the multidisciplinary committee when developing the new consensual language version ready for back-translation. In all age-specific versions of QUALAS, the questionnaire instructions and response options were well-received by the study participants.

Considering QUALAS-C, the two participating parents were generally satisfied with the Swedish translation but had concerns or suggestions for improving the clarity of five Swedish translated questions (Q1, Q2, Q3, Q8, Q10), three of which could be modified according to their preferences. They regarded the translations of “bother” and “health problem” (Supplementary Material S1). 

Teenagers with spina bifida gave suggestions to improve the Swedish translation of three questions (Q1, Q2, Q10) in QUALAS-T, two regarding the word “bother” and one the Swedish translation of “stool”. All were modified according to the study participant’s preferences. Additionally, the teenager expressed a need for higher literal consistency between the US English and Swedish translations of the response statements “I did not…” by removing words which had been added in Swedish. 

Adults with spina bifida provided much positive feedback on the Swedish translation of QUALAS-A, but expressed one main concern regarding Q8, “Did you worry about having children in the future, even if you already have a child?” One adult with spina bifida asked whether the original question referred to the equivalent Swedish translation “Did you worry about not being able to have children in the future?” The adult also suggested that “even if you already have a child” was not needed in the Swedish translation, because you can worry about this, whether or not you already have a child, and the question would be clearer using shorter wording. For consistency, the Swedish translation was adjusted accordingly in both QUALAS-A and QUALAS-T.

#### 3.1.4. Step 4—Back-Translation and Back-Translation Review

A back-translation of all age-specific versions of QUALAS was conducted by a professional translator who was a native US English speaker and who had not seen the original source language versions. The instrument developer compared all back-translated questionnaire instructions, questions and response options to QUALAS-C, QUALAS-T and QUALAS-A in US English with the original US English source versions, to ensure equivalence between the Swedish and the US English versions. The back-translation of the Swedish questionnaire instructions and response options to QUALAS-C, QUALAS-T and QUALAS-A showed good agreement with the original source language. The back-translation review discussion of the questions included in QUALAS-C, QUALAS-T and QUALAS-A are described in detail in Supplementary Material S1. Specifically, in QUALAS-C, eight questions were raised by the Swedish committee for discussion with the instrument developer, six questions in QUALAS-T and seven in QUALAS-A. Several questions concerned the same issue across the age-specific versions of QUALAS and included:

- attempts to ensure the equivalence of the Swedish–US English translation of “bother” used in and across QUALAS’ age-specific versions (QUALAS-C, Q3, Q4, Q5, Q7, Q8, Q10; QUALAS-T, Q1, Q2, Q7, Q9, Q10; QUALAS-A, Q7, Q12, Q14, Q15);
- removing the words “disposable underwear” in Swedish (QUALAS-C, Q6; QUALAS-T, Q6; QUALAS-A, Q11);
- the translation of “urine problems” vs. “bladder problems” (QUALAS-C, Q8, QUALAS-T, Q8; QUALAS-A, Q13);
- the strategy for age-appropriate wording of the questions included in the QUALAS-C, QUALAS-T and QUALAS-A, respectively. The participants did not always give the same input on the Swedish translations, which were similar in the three versions. This resulted in minor revisions in wording to achieve age-appropriate adjustments. One example is the way patients expressed, “stool to come out” in QUALAS-C (Q10) compared to QUALAS-T (Q10) and QUALAS-A (Q15). This varied, like the term “health problems” in QUALAS-C (Q2) and QUALAS-A (Q2, Q7, Q10). In this discussion, the instrument developer supported consistency in the wording of questionnaires used by different age groups, in order to enable the comparison of condition-specific HRQoL outcomes through life. Given this input, special attention was given by the Swedish project committee to creating linguistic consistency when developing the Swedish versions of QUALAS-C, QUALAS-T and QUALAS-A prior to cognitive debriefing (Supplementary Material S1).

All issues in the back-translation review were resolved. 

### 3.2. Cognitive Debriefing

#### 3.2.1. Step 5—Cognitive Debriefing Interviews

The Swedish translations of QUALAS, including the questionnaire instructions, response scales and overall evaluation, were generally reported as good by the respondents. It was also possible to identify and resolve issues with lack of clarity. A detailed presentation of the procedure, results for each of the questions, an expert discussion of the cognitive debriefing and, if judged needed, the proposed changes, is presented in Supplementary Material S2 separately for QUALAS-C, QUALAS-T and QUALAS-A. 

##### QUALAS-C

All children (*n* = 5, 100%) reported that the Swedish questionnaire instructions and response options were easy to understand. Table 3 presents the children’s evaluation of the translated Swedish questions in QUALAS-C during the cognitive debriefing which needed improvement in the translation. Four questions out of ten were rated as easy to understand by all five children. Only two questions needed a more thorough discussion and consideration by the Swedish project committee as a result of the children’s input in cognitive debriefing interviews.

- The US English question 1 (Q1) asked “Did you feel embarrassed about how you look?”, to which three children commented that they did not understand the Swedish translation of “embarrassed” (generad). Because of the children’s comments and the fact that the translation of “embarrassed” could be emotive in the Swedish language, the Swedish project committee decided to propose a change in the translation to a semantically equivalent expression in Swedish “Did you feel shy/insecure about how you look?” (Har du känt dig blyg/osäker över hur du ser ut?).
- The US English translation of question 2 (Q2) asked the child “Did dealing with health problems upset you?”. Four out of five children expressed difficulties in understanding the Swedish translation, as they did not understand the phrase “health problems” (*n* = 2). The suggested improvements included providing examples in brackets of what dealing with health problems could mean. The Swedish project committee considered this appropriate, as this would not change the word “health” and stay equivalent to the English version, while clarifying and concretizing the possible meaning in the Swedish translation.

None of the children regarded the questions as sensitive or uncomfortable to answer and of the three children who replied, all confirmed that the questions were adequate for the domains “Esteem and Independence” and “Bladder and Bowel”. None of the children expressed a need to remove or add any further questions to QUALAS-C. However, children’s comments on the questions revealed that it was sometimes difficult for them to rate their experience using a 5-point Likert response scale.

##### QUALAS-T

All teenagers (*n* = 6, 100%) reported the questionnaire instructions and response options as easy to understand. Table 4 presents the teenagers’ evaluations of the translated Swedish question 4 in QUALAS-T, which needed revision. All ten questions were rated as easy to understand by all teenagers, and eight questions explicitly received recognition and understanding, as seen in the study participants’ comments about them. However, the teenagers occasionally needed clarification by the interviewer to rate their experience on the 5-point Likert scale. In the Swedish translation of QUALAS-T, one change was deemed necessary based on teenagers’ evaluations. This regarded question 4 (Q4), which asked in US English “Did you worry about finding a boyfriend or girlfriend/husband or wife?”. Five out of six teenagers expressed difficulties understanding the Swedish translation. Based on the input from teenagers, the Swedish project committee suggested removing “husband and wife” as this was regarded as not applicable for teenagers in a Swedish setting. 

Two teenagers regarded a few questions within the domain “Family and independence” as sensitive/uncomfortable to answer (one teenager Q4, Q5; one teenager Q2), and one teenager regarded it as sensitive/uncomfortable to answer question 8 in the “Bladder and Bowel” domain.

The majority (*n* = 5, 83%) of the teenagers answered that the questions in the domain “Family and Independence” were adequate, and all of them (*n* = 6, 100%) answered that the questions in the domain “Bladder and Bowel” were similarly appropriate. None of the teenagers described a wish to remove or add further questions to the domains.

##### QUALAS-A

All adults (*n* = 5, 100%) reported the questionnaire instructions and response options as easy to understand. Table 5 presents the adults’ evaluation of the Swedish questions in QUALAS-A which needed improvement in wording. All translated questions in Swedish were rated as easy to understand by 80–100% of the adults and eleven questions received positive feedback for their recognition and understanding of the problems of adults with spina bifida. One change in the Swedish translation was judged necessary by the Swedish project committee due to comments made by the adults that the question was difficult to understand and sensitive/uncomfortable to answer (Q6, Table 5). Furthermore, there was a need for harmonizing the Swedish translation of “embarrassed” with that used in the revised Swedish translation of QUALAS-C (Q6).

One adult regarded it sensitive/uncomfortable to answer questions on “Health and relationships”, three about “Esteem and Sexuality”, but none of them regarded it sensitive/uncomfortable to answer questions in the “Bladder and Bowel” domain. 

All adults (*n* = 5, 100%) described the questions in the domains “Health and relationships”, “Esteem and Sexuality, and “Bladder and Bowel” as appropriate. None of them expressed a need to remove or add further questions to the domains. None of the adults reported a need to add questions to improve comprehensiveness of QUALAS-A.

### 3.3. Step 6—Production of the Final Swedish Language Version of QUALAS

Together with the original instrument developer of QUALAS, the Swedish translation of QUALAS-C, QUALAS-T and QUALAS-A was finalized and regarded as having achieved linguistic, content and face validity.

## 4. Discussion

In line with increased survival rates and improved prognosis for patients with spina bifida reaching adulthood [7,8], HRQoL has become increasingly important as an outcome measure used in clinical practice and in research [12,37]. This study reports the linguistic, content and face validity of the Swedish version of QUALAS-C, QUALAS-T and QUALAS-A for individuals with spina bifida, referencing condition-specific HRQoL instruments. Our process, which complies with a study protocol from the instrument developer, as well as with recommendations set forth by the US Food and Drug Administration [14], makes these assessments available for use in academic research and clinical practice in populations of Swedish patients with spina bifida. As the Swedish questionnaires and the US English source version reach conceptual equivalence, this study facilitates international studies and the pooling of HRQoL data, which is deemed critical to obtaining an adequate population size in rare diseases [31,32]. 

Forward-back translation is a resource-heavy but important process [38]. Since a language spoken within a country may reflect cultural norms and values, working with language is a key element in the establishment of linguistic, content and face validity of a HRQoL questionnaire. In fact, these aspects are essential to ensure PROM meets the psychometric criteria for current standards, and the many different types of validity and reliability in larger-scale field tests [14,35]. This is necessary to ensure that when claims are made regarding patients’ health, and these are based on patient-reported measurements, these instruments are trustworthy [14,39]. In our study, linguistic challenges identified in the Swedish translation of the QUALAS included the translation of “bother”, resulting in adapting the Swedish translation of the question. This meant that one word in US English needed to be split up into several different ones in Swedish. Another issue was that “disposable underwear” is not used in Sweden, making it necessary to omit this from the questions. Furthermore, the use of “health problems” was identified early on as a difficult expression to translate, especially for children. However, through back-translation and close collaboration with the instrument developer, such issues could be solved and/or possible solutions could be brought into the cognitive debriefing with patients. One example was the need to create consistency in the wording of similar items across QUALAS-C, QUALAS-T and QUALAS-A. Perhaps the careful collaborative multidisciplinary work, with attention paid to each of the translation steps, helped optimize the Swedish versions of QUALAS. The translation of an instrument into another language is a complex undertaking but a critical step in adopting an instrument for use in another country/language. While maintaining the semantical equivalence with the original source language instrument, it is not uncommon that the item wording needs to be culturally adapted to the normal speech patterns and colloquialisms of the target country/culture [31,38]. For example, in the Korean [33] and Japanese versions of QUALAS [26,27], questions regarding sexuality were very often not endorsed. Furthermore, in the Korean version of QUALAS: “two participants suggested that it was difficult to understand the content of “if the family treated you differently...”, as the meaning of “treated you differently” was unclear, resulting in the decision to add “special treatment or discrimination” to clarify the question. Pre-testing QUALAS-C, QUALAS-T and QUALAS-A by performing cognitive debriefing interviews has been vital in establishing and confirming face and content validity. At this stage of evaluation it is possible to adjust the translations before the questionnaire is administered to the target population (field test) [28,36]. Overall, in the cognitive debriefing, most questions were perceived as clear by the majority of study participants. The cognitive debriefing technique encompasses quantitative and qualitative inquiries [36], which were shown to be important in this study in detecting the difficulties study participants perceived in the translated questionnaires. This may be especially important to consider in HRQoL assessments of people with spina bifida, as many of them have impaired cognitive abilities [40,41,42], manifested in problems with language, perception, memory, and executive and attentional functions. For example, we observed some challenges for the children to rate their experience of HRQoL on a 5-point Likert scale. The use of a 5-point Likert scale in HRQoL questionnaires has generally been shown to work well in children from the age of 8 years, and with increased age, their health-related vocabulary grows [43,44]. In our study, even in interviews with teenagers, explanations or instructions on how to rate their experiences on the 5-point Likert scale were often needed. Interestingly, challenges encountered during translation also appeared in the cognitive debriefing of children. The two main issues regarded the understanding of “embarrassed” and “health problem”. “Health” represented a broad and abstract concept in the Swedish language, with a need for concretization for children. Interestingly, both in the teenage and in the adult study populations, the HRQoL questions in QUALAS were easily recognized, indicating they touched on familiar concepts. This was especially noted among the adult participants, whose interviews lasted much longer than the children’s, as the HRQoL questions seemed to trigger adults to share their HRQoL experiences. Facilitating the voices of people living with spina bifida by using interview techniques and giving them the possibility to express their experiences in their own words has previously been shown to add richness and trustworthiness to results [45,46].

Moreover, we asked the study participants if any question in QUALAS was sensitive/uncomfortable to answer. One reason for asking this question is that the perception of a question being sensitive/uncomfortable to answer could interfere with the openness of the reply and/or the wording used in the new translation could be emotive [38,47]. In our study, we found that none of the children found any question sensitive/uncomfortable to answer. Some teenagers and adults did report questions as sensitive/uncomfortable but these related to social aspects and sexuality rather than to bladder and bowel problems, and participants did not consistently point out the same questions. These results could indicate that study participants were relaxed and secure in discussing certain topics. As bladder and bowel problems are so common in patients with spina bifida [48], and the study participants were recruited from a cohort who took part in a urotherapy follow-up program, this could help explain these findings. On the other hand, although it may be advantageous to be interviewed by researchers who are not involved in the specific care/treatment, from the point of view of patients, these are previously unknown people asking intimate HRQoL questions, which may potentially make it more difficult to speak to them about social aspects and sexuality. At the same time, several studies of sexuality in people with spina bifida have underlined the importance of including this topic [49,50]. The authors did not identify any previous studies focusing on topics perceived as sensitive by patients with spina bifida. However, looking at the previous literature on children with congenital malformations, when measuring condition-specific patient-reported outcomes in patients with cleft lip and/or palate, a subgroup of 23% reported feeling upset or unhappy about their appearance or how they looked after completing CLEFT-Q [51]. In another study based on a condition-specific questionnaire for children born with esophageal atresia (EA) [47,52], items were generally not perceived as sensitive/uncomfortable to answer, but in certain countries questions about a child’s social exclusion due to EA were perceived as sensitive/uncomfortable to answer. And across 14 countries, the most sensitive/uncomfortable question, rated as such by 9.9% of the children, was the one asking if they experienced sadness due to their condition [47]. 

The long-term intention of the Swedish study of QUALAS is to pave the way for condition-specific HRQoL questionnaires to be used in encounters between health care providers and patients with spina bifida. In earlier studies, utilizing a HRQoL questionnaire in clinical practice with children has been shown to improve communication on psychosocial issues [53]. In a systematic review from 2021, the authors concluded that patient feedback, as expressed in outcome measurement, probably produces moderate improvements in communication between healthcare professionals and patients, and is also helpful in diagnosis and notation, disease control, and HRQoL [54]. Today’s standard of care and treatment of patients with spina bifida includes supporting these patients’ HRQoL and mental health. This suggests questions regarding HRQoL and mental health in patients with spina bifida are important to ask, as an entry to providing them with adequate health care and treatment [12,55]. 

Another valuable experience in our study was the work to achieve consistency in Swedish wording across common items in QUALAS-C, QAULAS-T and QUALAS-A. First, the cognitive debriefing findings suggested age-specific adjustments of the wording, for example the translation of “stool” within the domain “Bladder and Bowel”. Age adjustments were also recommended in PROMs for children [43,44]; PROMs covering different child and adult life trajectories have entered the field. However, including age adjustments may impede the possibility of evaluating HRQoL outcomes consistently across patient ages. In recent studies, urinary incontinence is a main contributor to poor HRQoL issues in children with spina bifida [18,19,21,24,29,30]. Fecal incontinence [18,30,56] is related to poor HRQoL outcomes, regardless of age and amount [30]. The possibility of evaluating outcomes across different age spans in the “Bladder and Bowel” domain is essential in patients with spina bifida, as the questionnaires previously utilized in the assessment of patient-reported outcomes were heterogeneous and sometimes non-validated [23]. 

As a last point, our study found that, despite the short length of QUALAS-C, QUALAS-T and QUALAS-A, the comprehensiveness of the items was reported as good by the study participants, which strengthens their content validity [28,36]. Our initial findings are also similar to other studies regarding translating and evaluating QUALAS-C, QUALAS-T and QUALAS-A, indicating that condition-specific HRQoL issues in spina bifida are conceptually equivalent in countries such as Sweden, the US, Korea, Japan and Brazil [21,24,26,27,29,33,34,57]. 

This study is strengthened by its collaborative multidisciplinary approach, which includes patients/parents and the instrument developer. Furthermore, one strength is the careful compliance with recommendations for patient-reported outcome measures. Our study is limited by small sample size. However, Sweden is a low-population country and we successfully recruited the suggested samples in cognitive debriefings. All the participants were recruited from one center, but this center offers tertiary care and has a large geographical uptake in Sweden. Furthermore, the study sample consisted of a good spread as to patient age and sex. As in many cases of MMC, the majority of study participants had shunt-treated hydrocephalus, and as this may indicate the severity of these patients’ clinical presentation [41,58], this should be taken into account when interpreting our results. Furthermore, more characteristics regarding the patients and their parents could have been collected to increase the understanding of the study results, such as their educational status. As this study has been conducted to pave the way for a larger-scaled field test of QUALAS, this could be improved in the next run. 

## 5. Conclusions

This study has established the linguistic, content and face validity of the Swedish version of a Quality-of-Life Assessment of people with spina bifida, (QUALAS-C, QUALAS-T and QUALAS-A). The Swedish questionnaires and the US English source version reached conceptual equivalence, while people with spina bifida reported the questionnaires as clear, adequate and comprehensive. This study therefore has enabled future research into the instrument’s further validity and reliability in a larger-scaled field test, making these assessments available for use in academic research and clinical practice in populations of Swedish patients with spina bifida, and facilitating international studies with the pooling of data. Close collaboration between the original instrument developer, a multidisciplinary team and people with spina bifida is necessary for this achievement.

## Figures and Tables

**Table 1:** Presentation of QUALAS-C, QUALAS-T and QUALAS-A.

QUALAS-C ^a^ (*n*, Questions)	QUALAS-T ^a^ (*n*, Questions)	QUALAS-A ^a^ (*n*, Questions)
		Health and Relationships (5)
Esteem and Independence (5)	Family and Independence (5)	Esteem and Sexuality (5)
Bladder and Bowel (5)	Bladder and Bowel (5)	Bladder and Bowel (5)

^a^ Recall period, the last four weeks; 5-point Likert response scale, and an additional option to respond that they did not experience the issues mentioned.

**Table 2:** Characteristics of study participants (*n* = 16) in the cognitive debriefing interviews of QUALAS.

	Total (*n* = 16)	Children (*n* = 5)	Teenagers (*n* = 6)	Adults (*n* = 5)
Male sex, *n* (%)	8 (50)	4 (80)	1 (17)	3 (60)
Median age years (min–maximum)	16 (8–33)	11 (8–11)	15.5 (14–16)	28 (18–33)
Shunt-treated hydrocephalus, *n* (%)	12 (75)	4 (80)	3 (50)	5 (100)
Wheelchair, *n* (%)	12 (75)	4 (80)	4 (67)	4 (80)
Clean intermittent catheterization *n* (%)	16 (100)	5 (100)	6 (100)	5(100)

**Table 3:** Cognitive debriefing results of the Swedish version of questions included in QUALAS-C with children (*n* = 5) and decision-making process to change wording of Swedish translation.

		Child Perceiving the Item as Clear	Qualitative Data		Project Committee
Questions (Q)	Original Question in US English	*n*, (%)	Type of Difficulties Encountered	Strength	Decision to Change the Wording of the Swedish Translation
Q1	Did you feel embarrassed about how you look?	3 (60)	The children do not understand the Swedish translation for “embarrassed”/the question (*n* = 3)	None mentioned	Har du känt dig generad över hur du ser ut? -> Har du känt dig blyg/osäker över hur du ser ut?
Q2	Did dealing with health problems upset you?	3 (60)	The children do not understand the word “health problem” *(n* = 2) Instead of answering one of the response options, the child answers “no” *(n* = 1) The child says it is difficult to recall 4 weeks ago (*n* = 1)	None mentioned	Har du blivit upprörd (ledsen/arg) av att ta hand om dina hälsoproblem? -> Har du blivit upprörd (ledsen/arg) av att ta hand om din hälsa (ex ta lavemang, RIKa, sköta korsett eller sjukgymnastik)?

**Table 4:** Cognitive debriefing results of the Swedish version of questions included in QUALAS-T with teenagers (*n* = 6) and decision-making process to change wording of Swedish translation.

		Teenagers’ Perception of the Item as Clear	Qualitative Data		Project Committee
Questions (Q)	Original Question in English	*n* (%)	Type Difficulties Encountered	Strength	Decision to Change the Wording of the Swedish Translation
Q4	Did you worry about finding a boyfriend or girlfriend/husband or wife?	6 (100)	Cannot relate to the situation (*n* = 1) The question was difficult to understand at first (*n* = 1) Needed clarification from the parent (*n* = 2) Difficult to use the response options (*n* = 1)	Can relate to the question (*n* = 1)	Har du oroat dig över att kunna hitta en pojkvän eller flickvän/man eller fru? -→ Har du oroat dig över att kunna hitta en pojkvän eller flickvän?

**Table 5:** Cognitive debriefing results of the Swedish version of questions included in QUALAS-A with adults (*n* = 5) and decision-making process to change wording of Swedish translation.

		Adults’ Perception of the Item as Clear	Qualitative Data		Project Committee
Questions (Q)	Original Question in US English	*n* (%)	Type Difficulties Encountered	Strength	Decision to Change the Wording of the Swedish Translation
Q6	Did you feel embarrassed about how you look?	3 (60)	The response options are difficult to use (*n* = 2) The adult asks for clarification of the question *(n* = 1) The question was sensitive (*n* = 1)		Har du känt dig generad över hur du ser ut? -> Har du känt dig blyg/osäker över hur du ser ut?

## Data Availability

The data that support the findings of this study are available from the corresponding author, upon reasonable request.

## Supplementary Materials

The following supporting information can be downloaded at: https://www.mdpi.com/article/10.3390/ijerph21050624/s1, Supplementary File S1: The complete translation procedure for QUALAS-C, QUALAS-T and QUALAS-A; Supplementary File S2: A detailed presentation of the procedure, results for each of the questions, expert discussion of the cognitive debriefing and if judged needed, the proposed changes are presented in Supplemental Material S2 separately for QUALAS-C, QUALAS-T and QUA-LAS-A.
