# Supplementary material for: Linguistic, Content and Face Validity of the Swedish Version of a Quality-of-Life Assessment for Children, Teenagers and Adults with Spina Bifida
Source: Int J Environ Res Public Health. 2024 May 15;21(5):624. doi: 10.3390/ijerph21050624 (PMC11121182; doi:10.3390/ijerph21050624)
Supplement: Supplementary file 1 [file ijerph-21-00624-s001.zip › Supplementary Material S1.pdf]

Supplementary File S1.

THE TRANSLATION PROCEDURE OF QUALAS-C ACCORDING TO A PRE-DEFINED PROTOCOL
QUALAS is copyright-protected. Use can be licensed from the original instrument developer, Dr. Konrad Szymanski (szymanko@iu.edu)

		STEP 1 - Two independent forward translations		STEP 2- Reconciliation		STEP 3- Patient/Parent feedback and Consensus new language version			STEP 4 –Back-translation and back-translation review			Consensus version to be tested in cognitive debriefing interviews
	**Source**	**Forward 1- by urologist native in Swedish and fluent in English**	**Forward 2- by urologist native in Swedish and fluent in English**	**If comments were made by the clinical and methodological committee regarding particular issues related to the forward-translations**	**Consensus new language version**	**Comments made by patient/parents**	**Any new adjustment according to patient/parents after discussion among the authors**	**Consensus new language version after patient/parent testing**	**Back-translation by a professional translator native in US English and fluent in Swedish**	**Back-translation review by local Swedish committee**	**Back-translation review by instrument developer**	**Consensus version to be tested in cognitive debriefing interviews**
	**Esteem and Independence**	Självkänsla och oberoende	Självkänsla och oberoende	OK	Självkänsla och självständighet	OK			Self-esteem and independence	OK	OK	Självkänsla och självständighet
**Q1**	Did you feel embarrassed about how you look?	Har du känt dig generad över hur du ser ut?	Skämdes du över hur du ser ut?	”embarrassed” refers literally to an emotive expression in Swedish and it may not be suitable to give the questionnaire that start, still the closest translation to the semantical meaning should be used	Har du känt dig generad över hur du ser ut?	Parents asked what the question referred to because the child would have responded differently if the question related to the face, the wheel-chair or the body.	No adjustement, cannot change because it does not comply with the original source question		Have you felt embarrassed about how you look?	OK	OK	Har du känt dig generad över hur du ser ut?
**Q2**	Did dealing with health problems upset you?	Har du blivit ledsen av att hantera dina hälsoproblem?	Blev du upprörd när du måste göra saker på grund av ditt funktionshinder?	“Health problem” is a difficult word in Swedish and not commonly used	Har du blivit ledsen eller arg av att ta hand om problem med din hälsa?	This question is difficult. What is a “health problem”? Health can be related to allergy, spina bifida or anything else.	Change to health related to spina bifida	Har du blivit ledsen eller arg av att ta hand om problem kopplat till ryggmärgsbråck/ ryggmärgsmissbildning?	Have you gotten sad or angry about dealing with problems connected with spina bifida occulta/cystica?	1) We believe the semantical meaning is practically the same, but the expression upset requires two words in Swedish. A disadvantage is that it may be understood as two questions, but this was not raised during the patient/parent review.	1) Which word do people consider, try to use only one if possible. Upset can include sad/angry/disappointed/other emotions.<br>2) The idea is to ask about health in	Har du blivit upprörd (ledsen/arg) av att ta hand om dina hälsoproblem?

Supplementary File S1.

				among children		Why not use health related to spina bifida?				2) Patient/parents felt that "health problems" is a broad term, and that respondents may mistakenly answer this not necessarily related spina bifida. Would it be ok from your perspective to use "problems connected with spina bifida"?	general (not health relating to spina bifida) for this question. The patient decides what issues they consider as important.	
	I did not have health problems	Jag har inte haft några hälsoproblem	Jag kände mig frisk	Health problem is a difficult word in Swedish and not commonly used among children	Jag har inte haft några problem med min hälsa	Same comment regarding health problem	Change to only problem	Jag har inte haft några problem	I haven't had any problems	OK	Needs to synchronize with the question, use the term "health problem"	Jag har inte haft några hälsoproblem.
**Q3**	Did it bother you to catheterize to get urine out?	Har du besvärats av att kissa med kateter?	Var det jobbigt att behöva använda kateter för att få ut urinen?	Adjust the Swedish language to clarify the meaning of "catheterize" in brackets	Har det varit jobbigt för dig att RIK:a dig (kissa med kateter)?	Parents expressed dissatisfaction with this question and asked about how it should be understood. The unclarity regarded whether the problem related to "entering the CIC-channel physically" or a problem if the child doesn't pee like other children? "Jobbigt" can be interpreted as difficult. "Känns det besvärligt…" and "Har det stört dig.." are better expressions.	Change to "stört dig"	Har det stört dig att du behövt RIK:a dig (kissa med kateter)?	Has it bothered you that you've had to self-catheterize (urinate with a catheter)?	The parents participating in the interview asked whether this question refers to possible physical problems to catheterize to get urine out or emotional/psychosocial problems, implicitly e.g., by not peeing like peers? Depending on which, we need to adapt the Swedish translation.	The question refers to any problems and all problems related to catheterizing (these are not specific to a particular area of life: physical, emotional, psychological, etc.). This is meant to be a general question that captures ALL the potentially affected areas -- the person answering it determines which problems apply or not. The back translated question seems to ask about being bothered by the NECESSITY of catheterizing (not catheterizing in general).	Har det känts besvärligt att använda RIK (kissa med kateter)?
	I did not catheterize	Jag använde inte kateter	*Jag använde inte kateter*	*OK*	*Jag har inte använt kateter*	OK			*I have not used a catheter*	OK	OK	Jag har inte använt kateter

Supplementary File S1.

**Q4**	Did it bother you if people in your family needed to help you?	Har det stört dig om någon i din familj behövt hjälpa dig?	Kändes det jobbigt om personer i din familj behövde hjälpa dig?	OK	Har det känts jobbigt om personer i din familj behövt hjälpa dig?	OK			Has it been difficult for you if people in your family had to help you?	OK	These questions should be phrased the same as that in QUALAS- T.	Har det stört dig om personer i din familj behövt hjälpa dig?
	I did not need help	Jag har inte behövt hjälp	Jag behövde ingen hjälp	OK	Jag har inte behövt någon hjälp	OK			I haven’t needed any help	OK	OK	Jag har inte behövt någon hjälp
**Q5**	Did it bother you if your friend or people outside your family needed to help you?	Har det stört dig om vänner eller någon utanför familjen behövt hjälpa dig?	Kändes det jobbigt om din vän eller andra människor utanför din familj behövde hjälpa dig?	Different words for ”friends” available in Swedish- the word needs to be adjusted for more common use	Har det känts jobbigt om din kompis eller någon utanför familjen behövt hjälpa dig?	OK			Has it been difficult for you if your friend or someone outside your family had to help you?	The word "bother" can be translated into different ways in Swedish depending on its contextal use. Is this ok?	OK	Har det stört dig om din kompis eller någon utanför familjen behövt hjälpa dig?
	I did not need help	Jag har inte behövt hjälp	Jag behövde ingen hjälp		Jag har inte behövt någon hjälp	OK			I haven’t needed any help	OK	OK	Jag har inte behövt någon hjälp
	**Bladder and Bowel**	Blåsa och tarm	Blåsa och tarm		Blåsa och tarm	OK			Bladder and bowel	OK	OK	Blåsa och tarm
**Q6**	Did you worry someone would notice your pads, pull-ups or disposable underwear?	Har du oroat dig för att någon skulle märka dina skydd, blöjor eller engångsunderkläder?	Oroades du över om någon skulle märka att du har skydd eller blöjor?	Clarify ”notice” in Swedish. There is no good translation in Swedish for “disposable underwear”- remove.	Har du oroat dig för att någon skulle lägga märke till dina skydd eller blöjor?	OK, good question			Have you worried that someone would notice your pads or diapers?	In Sweden, “disposable underwears” are not used. Therefore, this word is removed. OK?	Ok	Har du oroat dig för att någon skulle lägga märke till dina skydd eller blöjor?
	I did not wear any	Jag har inte använt några	Jag använde inga skydd		Jag har inte använt några skydd eller blöjor	OK			I haven’t used any pads or diapers	OK	OK	Jag har inte använt några skydd eller blöjor.

Supplementary File S1.

**Q7**	Did leaking urine bother you?	Har det stört dig att läcka urin?	Var du bekymrad över att läcka urin? (läcka kiss kanske är bättre)	OK	Har det bekymrat dig att läcka kiss?	OK			Have you been concerned about leaking pee?	The word bother is in this context translated to "concerned". We believe, as did the family representatives that, this was a good Swedish translation. Ok?	OK	Har det bekymrat dig att läcka urin?
	I did not leak	Jag har inte läckt	Jag läckte inte	There is a need to concretize leakage of what in a child-friendly way	Jag har inte läckt kiss	OK			I haven’t leaked pee	OK	OK, make sure it is the same wording across the age-specific versions of QUALAS	Jag har inte läckt urin.
**Q8**	Did your urine problems stop you from doing fun things?	Har dina urinproblem hindrat dig från att göra roliga saker?	Hindrades du från att göra roliga saker på grund av hur din urinblåsa fungerar?	OK	Har dina problem med urinblåsan hindrat dig från att göra roliga saker?	OK, good translation, and a big issue in the child’s life. The parents’ concern was if the child would understand the question and asked whether it was permitted to give a few examples.	Cannot change the wording because it does not comply with the original source question	Har dina problem med urinblåsan hindrat dig från att göra roliga saker?	Have your bladder problems prevented you from doing fun things?	Following the procedure, our suggestion is to translate urine problems to bladder problems because the literal expression of urine problems would sound strange in Swedish as judged by both clinical, methodological and family experiences.	The Swedish translation should be more equivalent with the US English translation and therefore keep “urine problems”. The reasons the question is phrased this way is because the item is supposed to include those patients who may not have or use their bladder can still answer the question. For example, these were the patients who have a urinary diversion (ileal conduit, etc.) -- and capture leaking urine from a stoma bag, etc. I think it is important to have a question that is answerable by all patients, regardless of how they manage their urine. Can you use some other phrasing? Urinary problems? Problems with your urine?	Har dina problem med urinen hindrat dig från att göra roliga saker?
	I did not have urine problems	Jag har inga problem med urinen	Jag hade inga problem med urinen	”Urine problem” need to clarify the wording in Swedish to reveal its context to the bladder	Jag har inga problem med urinblåsan	OK			I don’t have any bladder problems	Following the procedure, our suggestion is to translate urine problems to bladder problems because the literal expression of urine problems would sound strange in Swedish as judged by both clinical, methodological and family experiences. OK?	Similarly, this should reflect urine coming from places other the bladder as well	Jag har inga problem med urinen.
**Q9**	Did leaking stool bother you?	Har det stört dig att läcka avföring?	Stördes du över att läcka avföring?	Adjustment of ”bother” to a more semantical but not	Har det bekymrat dig att läcka avföring?	OK			Have you been concerned about leaking feces?	The word bother is in this context translated to "concerned". We believe, as did the family representatives that, this was a good Swedish translation. Ok?	Why not the same question for all age groups? As long as it is understood, that is enough.	Har det bekymrat dig att läcka avföring/bajs?

Supplementary File S1.

				literal meaning in Swedish								
	I did not leak	Jag har inte läckt	Jag läckte inte	OK	Jag har inte läckt	OK			I haven't leaked	OK	OK	Jag har inte läckt.
**Q10**	Did it bother you to wait for stool to come out?	Har det stört dig att vänta på att avföringen ska komma ut?	Stördes du över att behöva vänta på att avföringen skulle komma ut?	Adjust the Swedish language to clarify the meaning of "wait for stool to come out"	Har det stört dig att det tar lång tid på toaletten för att tömma tarmen?	OK, bother" has different meanings in Swedish – the best translation would be "Har det varit jobbigt för dig att det tar tid på toaletten när du ska bajsa"	Adjusted according to parents' preferences from "Stört dig" to "jobbigt" and from "tömma tarmen" to "bajsa"	Har det varit jobbigt för dig att det tar tid på toaletten när du ska bajsa?	Has it been difficult for you that it takes time in the bathroom when you have to poop?	1) Is the semantical meaning "takes time" correct?<br>2) The participants who provided input to the QUALAS-C, QUALAS -T and QUALAS A respectively, did not always make the same input to the Swedish translations being similar in the three versions. This practically means that we included minor revisions in wording. These minor revisions don't change the meaning of the question. We regard this as appropriate age-adjustments, but we want to make sure this complies with the study protocol? One example is that the way patients express "stool to come out" is different in QUALAS-C compared to QUALAS-T and QUALAS-A, ie for children compared to older age groups.<br>3) The word "bother" can be translated in different ways in Swedish, and in this context, it means has it been difficult or has it been hard for you?	1) Yes, takes time means delay, waiting -- the idea was to capture the negative impact of waiting due to constipation.<br>2) I see that the questions are quite different, and I'm not sure if they actually ask the same thing... The idea is to use the same question to be able to compare the same questions (and domain) across all ages. Why not use the same question for all age groups? It may not be the ideal for everyone, it might be good enough to work.<br>3) Bother is meant to capture the negative impact: why not use the same word as in Q14 or other times the word was used? Yes: the meaning is it has been difficult.	Har det stört dig att det tar tid på toaletten för avföringen/bajset att komma ut?
	I did not have to wait	Jag har inte behövt vänta	Jag behövde inte vänta	Adjustement of wording to more common use in Swedish	Det har inte tagit lång tid för mig	OK, adjust according to the question above	Adjusted according to parents' preferences of wording	Det har inte tagit lång tid för mig	It hasn't taken me a long time	OK		Jag har inte behövt lång tid på toaletten.
	Thank you!	Tack!	Tack	OK	Tack	OK		Tack	Thank you	OK		Tack!

Supplementary File S1.

THE TRANSLATION PROCEDURE OF QUALAS-t ACCORDING TO A PRE-DEFINED PROTOCOL

QUALAS is copyright-protected. Use can be licensed from the original instrument developer, Dr. Konrad Szymanski (szymanko@iu.edu)

		**STEP 1 - Two independent forward translations**		**STEP 2- Reconciliation**		**STEP 3- Patient/Parent feedback and Consensus new language version**			**STEP 4 -Back-translation and back-translation review**			**Consensus version to be tested in cognitive debriefing interviews**
	**Source**	**Forward 1- by urologist native in Swedish and fluent in English**	**Forward 2- by urologist native in Swedish and fluent in English**	**If comments were made by the clinical and methodological committee regarding particular issues related to the forward-translations**	**Consensus new language version**	**Comments made by patient/parents**	**Any new adjustment according to patient/parents after discussion among the authors**	**Consensus new language version after patient/parent testing**	**Back-translation by a professional translator native in US English and fluent in Swedish**	**Back-translation review by local Swedish committee**	**Back-translation review by instrument developer**	**Consensus version to be tested in cognitive debriefing interviews**
	**Family and Independence**	Familj och oberoende	Familj och oberoende	OK	Familj och Självständighet	OK		Familj och Självständighet	Family and independence	Ok	**OK**	Familj och självständighet
**Q1**	Did it bother you if people in your family needed to help you?	Har det stört dig om någon i din familj behövt hjälpa dig?	Störde det dig om dina familjemedlemmar behövde hjälpa dig?	”Bother you” is a complex word in the Swedish language. In this context, it may be better to adjust the wording to a more commonly used wording.	Har du tyckt att det varit jobbigt om någon i din familj behövt hjälpa dig?	“Bother”, means if it has disturbed you, and not that anything is difficult for you. The meaning of “Bother“ means that something is disturbing- change the translation.	”Har det stört dig om en familjemedlem behövt hjälpa dig?”.	”Har det stört dig om en familjemedlem behövt hjälpa dig?”.	“Has it bothered you if a family member had to help you?”	OK	Are these questions phrased the same as in QUALAS-C-they should be to keep the consistency across the age-specific versions of QUALAS.	Har det stört dig om personer i din familj behövt hjälpa dig?
	I did not need help	Jag har inte behövt hjälp	Jag behövde ingen hjälp"		Jag har inte behövt någon hjälp	OK		Jag har inte behövt någon hjälp	I haven’t needed any help	OK	OK	Jag har inte behövt någon hjälp.

Supplementary File S1.

**Q2**	Did it bother you if your friend or people outside your family needed to help you?	Har det stört dig om din vän eller någon utanför familjen behövt hjälpa dig?	Störde det dig om din vän eller någon utanför familjen behövde hjälpa dig?	1) "Bother you" is a strong and emotive expression in Swedish. Needs to be adjusted to the context of use.<br><br>2) The word "Vän" is not commonly used among children/teenagers. Adjust.	Har du tyckt att det varit jobbigt om din kompis eller någon utanför familjen behövt hjälpa dig?	Same comment regarding "bother you" as before. Bother means something is disturbing, but it doesnt`t have to be troublesomeand it is not the same as annoying. Change to a better literal translation.	"Har det stört dig om din kompis eller någon utanför familjen behövt hjälpa dig?"	"Har det stört dig om din kompis eller någon utanför familjen behövt hjälpa dig?"	"Has it bothered you if your friend or someone outside the family had to help you?"	OK	Are these questions phrased the same as in QUALAS-C-they should be to keep the consistency across the age-specific versions of QUALAS.	Har det stört dig om din kompis eller någon utanför familjen behövt hjälpa dig?
	I did not need help	Jag har inte behövt hjälp	Jag behövde ingen hjälp			OK		Jag har inte behövt någon hjälp	I haven't needed any help	OK	OK	Jag har inte behövt någon hjälp.
Q3	Did it annoy you if you could not do what other teenagers could do?	Har det irriterat dig om du inte kunnat göra vad andra tonåringar kan göra?	Blev du irriterad om du inte kunde göra sådant som andra tonåringar kan göra?		Har det irriterat dig om du inte kunnat göra vad andra tonåringar kan göra?	OK		Har det irriterat dig om du inte kunnat göra vad andra tonåringar kan göra?	Has it annoyed you if you haven't been able to do what other teenagers can do?	OK	OK	Har det irriterat dig om du inte kunnat göra vad andra tonåringar kan göra?
	I could do what other teens do	Jag kan göra vad andra tonåringar gör	Jag kunde göra vad andra tonåringar gör		Jag kan göra samma saker som andra tonåringar	OK		Jag kan göra samma saker som andra tonåringar	I can do the same things as other teenagers	OK	OK	Jag kan göra samma saker som andra tonåringar
Q4	Did you worry about finding a boyfriend or girlfriend / husband or wife?	Har du oroat dig över att hitta en pojkvän eller flickvän / man eller fru?	Oroade du dig över att inte få en pojkvän eller flickvän/man eller fru?	The wording in Swedish is difficult because it can be interpreted that they should worry about finding boy-or girlfriend or be concerned of it.	Har du oroat dig över att kunna hitta en pojkvän eller flickvän / man eller fru?	OK		Har du oroat dig över att kunna hitta en pojkvän eller flickvän / man eller fru?	Have you worried about being able to find a boyfriend or girlfriend / husband or wife?	OK	OK	Har du oroat dig över att kunna hitta en pojkvän eller flickvän / man eller fru?

Supplementary File S1.

	I already have one	Jag har redan en	Jag har redan en		Jag har redan en	OK		Jag har redan en	I already have one	OK	OK	Jag har redan en
Q5	Did you worry about having children in the future, even if you already have a child?	Har du oroat dig över att få barn i framtiden, även om du redan har barn?	Kände du oro över att kunna få barn i framtiden även om du redan har barn?	1) Need to adjust the question ”even if you already have a child” to its relevance in Swedish setting, because it is rare that teenagers 13-17 with spina bifida already have a child.<br><br>2) Separate the question into two	Har du oroat dig över att kunna få barn i framtiden? Har du oroat dig för detta, även om du redan har barn?	OK	Remove “even if you have a child” in consistency with QUALAS-A.	Har du oroat dig över att inte kunna få barn i framtiden?	Have you worried about not being able to have children in the future?	Inn line with comments by the adult study participant the following issue needs clarification;<br><br>1) does the original question “Did you worry about having children in the future” be equivalent with the Swedish translation “Did you worry about not being able to have children in the future”? Or does the original question refer to “having children and being able to care for them”?<br><br>2) There is also a need to remove the part "even if you already have a child, as in Sweden this patient group usually does not become a parent during teenage years.	1) The original question was meant to capture concerns about fertility. The actual question (as your patients accurately point out) also captures the ability taking care of a child making this a general question.<br><br>2) If you need to settle on one meaning, aim for the first one (fertility). During our pilot testing, patients asked, specifically: what if I already have a child? -- that is why we added this. The Swedes may think differently, so if you think this is not needed, it would be ok to remove that phrase. I agree with your reasoning.<br><br>If this phrase is dropped, I would ensure there is a "cost" to keeping it -- people get weirded out or confused, and no benefit in keeping it (Swedish speaking people with spina bifida who have children already won't need this clarification that the question also applies to them).	Har du oroat dig över att inte kunna få barn i framtiden?
	**Bladder and Bowel**	Blåsa och tarm	Blåsa och tarm	OK	Blåsa och tarm	OK		Blåsa och tarm	Bladder and bowel	OK		Blåsa och tarm
Q6	Did you worry someone would notice your pads, pull-ups or disposable underwear?	Har du oroat dig för att någon skulle märka dina skydd, blöjor eller engångsunderkläder?	Var du orolig för att någon skulle upptäcka att du har kontinensskydd eller blöjor?	Clarify ”notice” in Swedish. There is no good translation in Swedish for “disposable underwear”- remove.	Har du oroat dig för att någon skulle lägga märke till dina skydd eller blöjor?	Ok		Har du oroat dig för att någon skulle lägga märke till dina skydd eller blöjor?	Have you worried that someone would notice your pads or diapers?	In Sweden, “disposable underwears” are not used. Therefore, this word is removed. OK?	OK	Har du oroat dig för att någon skulle lägga märke till dina skydd eller blöjor?
	I did not wear any	Jag har inte använt några	Jag använde inga	OK	Jag har inte använt några	Feels like words have been added that are	Remove “skydd	Jag har inte använt några	I haven’t used any	OK	OK, make sure it is the same wording across the age-specific versions of QUALAS	Jag har inte använt några skydd eller blöjor.

Supplementary File S1.

					skydd eller blöjor	part of the original statement- remove.	eller blöjor"					
Q7	Did leaking urine bother you?	Har det stört dig att läcka urin?	Bekymrade du dig över att läcka urin?	Use the same tempus and wording in all age-specific versions	Har det bekymrat dig att läcka urin?	This translation of "Bother" is ok to use in this sentence		Har det bekymrat dig att läcka urin?	Have you been concerned about leaking urine?	The word "bother" is in this context translated to "concerned". We believe, as did the patient representatives that, this was a good Swedish translation.	OK	Har det bekymrat dig att läcka urin?
	I did not leak	Jag har inte läckt	Jag läckte inte	Need to concretize leakage of what	Jag har inte läckt urin	OK, need remove words that have been added.	Remov e "urin"	Jag har inte läckt	OK		OK, make sure it is the same wording across the age-specific versions of QUALAS	Jag har inte läckt.
Q8	Did your urine problems stop you from doing fun things?	Har dina urinproblem hindrat dig från att göra roliga saker?	Hindrades du från att göra roliga saker på grund av ditt blåsproblem ?	Does the word "urine problems" refer to the leakage or the whole situation including clean intermittent catheterization? Merge the two suggestions into "problem med urinblåsan"	Har dina problem med urinblåsan hindrat dig från att göra roliga saker?	Ok		Har dina problem med urinblåsan hindrat dig från att göra roliga saker?	Have your bladder problems prevented you from doing fun things?	Following the procedure, our suggestion is to translate urine problems to bladder problems because the literal expression of urine problems would sound strange in Swedish as judged by both clinical, methodological and family experiences.	The Swedish translation should be more equivalent with the US English translation and therefore keep "urine problems". The reasons the question is phrased this way is because the item is supposed to include those patients who may not have or use their bladder can still answer the question. For example, these were the patients who have a urinary diversion (ileal conduit, etc.) -- and capture leaking urine from a stoma bag, etc. I think it is important to have a question that is answerable by all patients, regardless of how they manage their urine. Can you use some other phrasing? Urinary problems? Problems with your urine?	Har dina problem med urinen hindrat dig från att göra roliga saker?
	I did not have urine problems	Jag har inga problem med urinen	Jag hade inga problem med urinen	Adjust the Swedish language to clarify meaning of urine problems.	Jag har inga problem med urinblåsa	Ok		Jag har inga problem med urinblåsa	I don't have any bladder problems.	Following the procedure, our suggestion is to translate urine problems to bladder problems because the literal expression of urine problems would sound strange in Swedish as judged by both clinical, methodological and family experiences. OK?	Similarly, this should reflect urine coming from places other the bladder as well.	Jag har inga problem med urinen.

Supplementary File S1.

Q9	Did leaking stool bother you?	Har det stört dig att läcka avföring?	Var du bekymrad över att läcka avföring?	Adjustment of "bother" to a more semantical but not literal meaning in Swedish.	Har det bekymrat dig att läcka avföring?	OK		Har det bekymrat dig att läcka avföring?	Have you been concerned about leaking feces?	The word bother is in this context translated to "concerned". We believe, as did the family representatives that, this was a good Swedish translation. Ok?	OK	Har det bekymrat dig att läcka avföring/bajs?
	I did not leak	Jag har inte läckt	Jag läckte inte	OK	OK Jag har inte läckt	OK		Jag har inte läckt	I haven't leaked	OK	OK	Jag har inte läckt.
Q10	Did it bother you to wait for stool to come out?	Har det stört dig att vänta på att avföringen ska komma ut?	Bekymrade det dig att behöva vänta på att få ut avföringen?	Adjust the Swedish language to clarify the meaning of "wait for stool to come out"	Har det stört dig att det tar lång tid på toaletten för att tömma tarmen?	To "empty bowel" … Different sentence compared to the original source. Suggest adjustment.	Har det stört dig att det tar tid på toaletten för avföring att komma ut?	Har det stört dig att det tar tid på toaletten för avföring att komma ut?	Has it bothered you that it takes time on the toilet for feces to come out?	1. Is the semantical meaning "takes time" correct?<br>2. The participants who provided input to the QUALAS-C, QUALAS -T and QUALAS A respectively, did not always make the same input to the Swedish translations being similar in the three versions. This practically means that we included minor revisions in wording. These minor revisions don't change the meaning of the question. We regard this as appropriate age-adjustments, but we want to make sure this complies with the study protocol? One example is that the way patients express "stool to come out" is different in QUALAS-C compared to QUALAS-T and QUALAS-A, ie for children compared to older age groups.<br>3. The word "bother" can be translated in different ways in Swedish, and in this context, it means has it been difficult or has it been hard for you?	1. Yes, takes time means delay, waiting -- the idea was to capture the negative impact of waiting due to constipation.<br>2. I see that the questions are quite different, and I'm not sure if they actually ask the same thing... The idea is to use the same question to be able to compare the same questions (and domain) across all ages. Why not use the same question for all age groups? It may not be the ideal for everyone, it might be good enough to work.<br>3. Bother is meant to capture the negative impact: why not use the same word as in Q14 or other times the word was used? Yes: the meaning is it has been difficult.	Har det stört dig att det tar tid på toaletten för avföringen/bajset att komma ut?
	I did not have to wait	Jag har inte behövt vänta	Adjust the tempus in line with the other statements	Jag behövde inte vänta	It hasn´t taken long time for me.	The translation works, but I want to suggest a	Det har inte tagit lång tid för mig	Det har inte tagit lång tid för mig	It hasn't taken me a long time	OK	OK, make sure it is the same wording across the age-specific versions of QUALAS	Jag har inte behövt lång tid på toaletten.

Supplementary File S1.

						better translation.							
	Thank you!	Tack!	Tack	Tack	Ok	OK	Tack	Tack	Thank you	OK			Tack!

Supplementary File S1.

THE TRANSLATION PROCEDURE OF QUALAS-A ACCORDING TO A PRE-DEFINED PROTOCOL

QUALAS is copyright-protected. Use can be licensed from the original instrument developer, Dr. Konrad Szymanski (szymanko@iu.edu)

		**STEP 1 - Two independent forward translations**		**STEP 2- Reconciliation**		**STEP 3- Patient/Parent feedback and Consensus new language version**			**STEP 4 -Back-translation and back-translation review**			**Consensus version to be tested in cognitive debriefing interviews**
	**Source**	**Forward 1- by urologist native in Swedish and fluent in English**	**Forward 2- by urologist native in Swedish and fluent in English**	**If comments were made by the clinical and methodological committee regarding particular issues related to the forward-translations**	**Consensus new language version**	**Comments made by patient/parents**	**Any new adjustment according to patient/parents after discussion among the authors**	**Consensus new language version after patient/parent testing**	**Back-translation by a professional translator native in US English and fluent in Swedish**	**Back-translation review by local Swedish committee**	**Back-translation review by instrument developer**	**Consensus version to be tested in cognitive debriefing interviews**
	**Health and Relationship**	Hälsa och relationer	Hälsa och relationer		Hälsa och relationer	OK		Hälsa och relationer	Health and relationships	OK	OK	Hälsa och relationer
Q1	Were you happy with your health?	Har du varit nöjd med din hälsa	Var du nöjd med din hälsa?		Har du varit nöjd med din hälsa?	OK		Har du varit nöjd med din hälsa?	Have you been satisfied with your health?	Satisfied instead of happy is used in the back-translation.	OK	Har du varit nöjd med din hälsa?
Q2	Did you feel people see you as a person and not only your health problems?	Har du känt att folk ser dig som en person och inte bara dina hälsoproblem	Upplevde du att människor ser dig som en person och inte bara som någon med funktionshinder?	Using the term change ”health problems” in Swedish makes the question difficult. Adjust to a more semantical but not literal meaning in Swedish.	Har du känt att andra människor ser dig som en person och inte bara någon med hälsoproblem ?	It is good not to ask about not only becoming your health problems.		Har du känt att andra människor ser dig som en person och inte bara någon med hälsoproblem?	Have you felt that other people see you as a person and not just someone with health problems?	OK	OK	Har du känt att andra människor ser dig som en person och inte bara någon med hälsoproblem?
Q3	Were you able to do fun things	Har du kunnat göra roliga saker?	Hade du möjlighet att göra roliga saker?		Har du kunnat göra roliga saker?	OK		Har du kunnat göra roliga saker?	Have you been able to do fun things?	OK	OK	Har du kunnat göra roliga saker?

Supplementary File S1.

Q4	Did you spend time with your friends your age outside of school or work?	Har du spenderat tid med dina vänner i din ålder förutom i skolan eller på jobbet?	Tillbringade du tid med dina vänner i din egen ålder efter skolan eller arbetet?	Needs to be adjusted in Swedish language by removing ”your” in ”your friends”	Har du umgåtts med vänner i din egen ålder förutom i skolan eller på jobbet?	On-spot		Har du umgåtts med vänner i din egen ålder förutom i skolan eller på jobbet?	Have you spent time with friends your own age apart from in school or at work?	OK	OK	Har du umgåtts med vänner i din ålder förutom i skolan eller på jobbet?
Q5	Were you comfortable with your close friendships outside of your family?	Har du varit bekväm med dina nära vänner utanför din familj?	Var du nöjd med dina vänskapsrelationer som du har utanför familjen?		Har du varit nöjd med dina vänskapsrelationer som du har utanför familjen?	Translation is ok. The answer will be different in the pandemic time period.		Har du varit nöjd med dina vänskapsrelationer som du har utanför familjen?	Have you been satisfied with your friendships you have outside the family?	OK	OK	Har du varit nöjd med dina vänskapsrelationer som du har utanför familjen?
	**Esteem and Sexuality**	Självkänsla och sexualitet	Självkänsla och sexualitet		Självkänsla och sexualitet	OK, Translation good.		Självkänsla och sexualitet	Self-esteem and sexuality	OK	OK	Självkänsla och sexualitet
Q6	Did you feel embarrassed about how you look?	Har du känt dig generad över hur du ser ut?	Var du generad över hur du ser ut?	Discussion about the meaning of the question “how you look” or if it refers to “how your body looks” in Swedish language.	Har du känt dig generad över hur du ser ut?	Good translation		Har du känt dig generad över hur du ser ut?	Have you felt embarrassed about how you look?	OK	OK	Har du känt dig generad över hur du ser ut?
Q7	Did it bother you if your family treated you differently because of your health problems?	Har det störd tid om din familj behandlat dig annorlunda på grund av dina hälsoproblem?	Störde det dig om din familj behandlade dig annorlunda på grund av ditt funktionshinder?	There is a need to change the Swedish translation for ”health problems”. Adjust to a more semantical but not literal meaning in Swedish.	Har det stört dig om din familj behandlat dig annorlunda på grund av dina hälsoproblem?	OK, good		Har det stört dig om din familj behandlat dig annorlunda på grund av dina hälsoproblem?	Has it bothered you if your family treated you differently because of your health problems?	OK	OK	Har det störd dig om din familj behandlat dig annorlunda på grund av dina hälsoproblem?
	I was not treated differently	Jag har inte blivit annorlunda behandlad	Jag behandlades inte annorlunda		Jag har inte blivit annorlunda behandlad			Jag har inte blivit annorlunda behandlad	I haven’t been treated differently	OK	OK	Jag har inte blivit annorlunda behandlad.

Supplementary File S1.

Q8	Did you worry about having children in the future, even if you already have a child?	Har du oroat dig över att få barn i framtiden, även om du redan har barn?	Oroande du dig över att kunna få barn i framtiden även om du redan har barn?		Har du oroat dig över att kunna få barn i framtiden, även om du redan har barn?	It´s not needed in Swedish to ask ”even if you have a child”. The participant asked, if the original question referred to “Did you worry about having children in the future” equivalent in Swedish “Did you worry about not being able to have children in the future”? Or does the question “Did you worry about having children in the future” equivalent in Swedish to “having them and caring for them”? The adult with SB also found that “even if you already have a child" wasn't needed, because you can worry about this	Remove “even if you have a child”.	Har du oroat dig för att inte kunna få barn i framtiden?	Have you worried about not being able to have children in the future?	We decided to remove "even if you already have a child” due to patient input.	During our pilot testing, patients asked, specifically: what if I already have a child? -- that is why we added this. The Swedes may think differently, so if you think this is not needed, it would be ok to remove that phrase. I agree with your reasoning.<br><br>Fertility/birth issues are the primary intention, taking care of/raising children is a secondary aim. If you can, phrase the question to capture fertility, and if it still captures raising a child (great), but fertility was the main point.	Har du oroat dig över att inte kunna få barn i framtiden?

Supplementary File S1.

						independent of already having a child or not and that the question would be better in shorter wording.						
	No answer	Inget svar	Inget svar		Inget svar	OK		Inget svar	No answer	OK	OK	Inget svar
Q9	Were you happy with your sex life?	Har du varit nöjd med ditt sexliv?	Var du nöjd med ditt sexliv?		Har du varit nöjd med ditt sexliv?	OK	Har du varit nöjd med ditt sexliv?	Har du varit nöjd med ditt sexliv?	Have you been satisfied with your sex life?	OK	OK	Har du varit nöjd med ditt sexliv?
	No answer	Inget svar	Inget svar	Inget svar		OK		Inget svar	No answer	OK		Inget svar
Q10	Did you think your health problems will affect how satisfied you will be with sexual activity in the future?	Har du tänkt att dina hälsoproblem kommer att påverka hur nöjd du kommer vara med ditt sexliv i framtiden?	Trodde du att ditt funktionshinder kommer att påverka hur nöjd du kommer att vara med din sexuella aktivitet i framtiden?	Adjust the Swedish language to clarify the meaning of "health problems." Merge the two forward translations into one.	Har du tänkt att dina hälsoproblem kommer att påverka hur nöjd du kommer vara med din sexuella aktivitet i framtiden?	Consider the translation in Swedish "may affect" The question is very long, but can be understood.		Har du tänkt att dina hälsoproblem kan komma att påverka hur nöjd du kommer vara med din sexuella aktivitet i framtiden?	Have you thought that your health problems may affect how satisfied you will be with your sexual activity in the future?	OK	OK	Har du tänkt att dina hälsoproblem kan komma att påverka hur nöjd du kommer vara med din sexuella aktivitet i framtiden?
	No answer	Inget svar	Inget svar		Inget svar	OK		Inget svar	No answer	OK	OK	Inget svar
	**Bladder and Bowel**	Blåsa och tarm	Blåsa och tarm		Blåsa och tarm	OK		Blåsa och tarm	Bladder and bowel	OK	OK	Blåsa och tarm
Q11	Did you worry someone would notice your pads, pull-ups or disposable underwear?	Har du oroat dig för att någon skulle märka dina skydd, blöjor eller engångsunderkläder?	Var du orolig för att någon skulle upptäcka att du har kontinensskydd eller blöjor?	Clarify "notice" in Swedish. There is no good translation in Swedish for "disposable	Har du oroat dig för att någon skulle lägga märke till dina skydd eller blöjor?	Good translation.		Har du oroat dig för att någon skulle lägga märke till dina skydd eller blöjor?	Have you worried that someone would notice your pads or diapers?	In Sweden, "disposable underwears" are not used. Therefore, this word is removed. OK?	OK	Har du oroat dig för att någon skulle lägga märke till dina skydd eller blöjor?

Supplementary File S1.

				underwear”-remove.								
	I did not wear any	Jag har inte använt några	Jag använde inga	OK, add pads or diapers in Swedish language to make the statement more concrete	Jag har inte använt några skydd eller blöjor	Good translation.		Jag har inte använt några skydd eller blöjor	I haven’t used any pads or diapers	OK	OK	Jag har inte använt några skydd eller blöjor
Q12	Did leaking urine bother you?	Har det stört dig att läcka urin?	Bekymrade du dig över att läcka urin?	OK	Har det bekymrat dig att läcka urin?	OK		Har det bekymrat dig att läcka urin?	Have you been concerned about leaking urine?	The word “bother” is in this context translated to "concerned", and preferred also by the patient representatives as a good Swedish translation.<br><br>Make sure it is the same wording across the age-specific versions of QUALAS	OK	Har det bekymrat dig att läcka urin?
	I did not leak	Jag har inte läckt	Jag läckte inte	OK	Jag har inte läckt urin	OK		Jag har inte läckt urin	I haven’t leaked urine	OK	OK	Jag har inte läckt
Q13	Did your urine problems stop you from doing fun things?	Har dina urinproblem hindrat dig från att göra roliga saker?	Hindrades du från att göra roliga saker på grund av dina blåsproblem?	Does the word urine problems refer to the leakage or the whole situation including clean intermittent catheterization? Merge the two suggestions into “problem med urinblåsan”	OK	OK, good translation. Urine problems would be a strange translation because everyone who has these worries understand it´s impact. I prefer what we have written “problem med urinblåsan”		Har dina problem med urinblåsan hindrat dig från att göra roliga saker?	Have your bladder problems prevented you from doing fun things?	Following the procedure, our suggestion is to translate “urine problems” to bladder problems because the literal expression of urine problems would sound strange in Swedish as judged by both clinical, methodological and family experiences.	The Swedish translation should be more equivalent with the US English translation and therefore keep “urine problems”. The reasons the question is phrased this way is because the item is supposed to include those patients who may not have or use their bladder can still answer the question. For example, these were the patients who have a urinary diversion (ileal conduit, etc.) -- and capture leaking urine from a stoma bag, etc. I think it is important to have a question that is answerable by all patients, regardless of how they manage their urine. Can you use some other phrasing? Urinary problems? Problems with your urine?	Har dina problem med urinen hindrat dig från att göra roliga saker?

Supplementary File S1.

	I did not have urine problems	Jag har inga problem med urinen	Jag hade inga problem med urinen	OK	Jag har inga problem med urinen	Bladder, it should be consistent.	Adjust the word to bladder.	Jag har inga problem med urinblåsan	I don't have any bladder problems	Following the procedure, our suggestion is to translate "urine problems" to bladder problems because the literal expression of urine problems would sound strange in Swedish as judged by both clinical, methodological and family experiences.	See comment above. Change accordingly.	Jag har inga problem med urinen
Q14	Did leaking stool bother you?	Har det stört dig att läcka avföring?	Var du bekymrad över att läcka avföring?	Adjustment of "bother" to a more semantical but not literal meaning in the Swedish language	Har det stört dig att läcka avföring?	Good translation			Has it bothered you to leak feces?	OK	Make sure it is the same wording across the age-specific versions of QUALAS	Har det bekymrat dig att läcka avföring/bajs?
	I did not leak	Jag har inte läckt	Jag läckte inte	OK	Jag har inte läckt	Add "feces", should be consistent with these items.			I haven't leaked	OK	OK	Jag har inte läckt
Q15	Did it bother you to wait for stool to come out?	Har det stört dig att vänta på att avföringen ska komma ut?	Bekymrade det dig att behöva vänta på att få ut avföringen?	Har det stört dig att det tar lång tid på toaletten för att tömma tarmen?	The expression "stool to come out" is not commonly used among children, teenagers and adults in Sweden. Possible change to "tömma tarmen" in QUALAS-C, QUALAS -T and QUALAS A.<br>This change should not make an impact of the translation to English.	Good translation, captured the English content, understood the meaning of the question.		Har det stört dig att det tar tid på toaletten för att tömma tarmen?	Has it bothered you that it takes time on the toilet to have a bowel movement?	4) We want to ensure that the semantical meaning "takes time" is correct?<br>5) As written previously, the participants who provided input to the QUALAS-C, QUALAS -T and QUALAS A respectively, did not always make the same input to the Swedish translations being similar in the three versions. This practically means that we included minor revisions in wording. These minor revisions don't change the meaning of the question. We regard this as appropriate age-adjustments, but we want to make sure this complies with the study protocol? One example is that the way patients express "stool to come out" is different in QUALAS-C compared to QUALAS-T and	1. Yes, "takes time" means delay, waiting -- the idea was to capture the negative impact of waiting due to constipation.<br>2. I see that the questions are quite different, and I'm not sure if they actually ask the same thing. The idea is to use the same question to be able to compare the same questions (and domain) across all ages.<br>3. Bother is meant to capture the negative impact: why not use the same word as in Q14 or other times the word was used? Yes: the meaning is it has been difficult.	Har det stört dig att det tar tid på toaletten för avföringen/bajset att komma ut?

Supplementary File S1.

										QUALAS-A, ie for children compared to older age groups.<br><br>6) The word "bother" can be translated in different ways in Swedish, and in this context, it means has it been difficult or has it been hard for you... Wat do you think?		
	I did not have to wait	Jag har inte behövt vänta	Jag behövde inte vänta	OK, but add the word ”bathroom” in Swedish to make the statement more concrete	Jag har inte behövt ha lång tid på toaletten	Good translation.		Jag har inte behövt ha lång tid på toaletten	I haven't needed a long time on the toilet	OK	Keep the wording similar wording across the age-specific versions	Jag har inte behövt lång tid på toaletten
