# Supplementary material for: Linguistic, Content and Face Validity of the Swedish Version of a Quality-of-Life Assessment for Children, Teenagers and Adults with Spina Bifida
Source: Int J Environ Res Public Health. 2024 May 15;21(5):624. doi: 10.3390/ijerph21050624 (PMC11121182; doi:10.3390/ijerph21050624)
Supplement: Supplementary file 1 [file ijerph-21-00624-s001.zip › Supplementary Material S2.pdf]

Supplementary File S2.

Cognitive debriefing results of the Swedish version of questions included in QUALAS-C with children (n=5) and decision-making process to change wording of Swedish translation							
		**Child perceiving the item as clear**	**Qualitative data**		**Project committee**		
**Esteem and Independence**							
**Questions (Q)***	**Original question in English**	**n, (%)**	**Type difficulties encountered**	**Strength**	**Discussion about possible change**	**Discussion and consideration after the children's input**	**Decision to change the wording of the Swedish translation**
**Q1**	Did you feel embarrassed about how you look?	3(60)	The children do not understand the Swedish translation for "embarrassed"/the question (n=3)		Yes	The word "embarrassed" in Swedish is not commonly used among children. "Embarrassed" is a strong word in Swedish, and the word "embarrassed" about their looks could be perceived as emotive. Another translation was suggested with milder and child-adapted expressions such as feeling "shy/insecure about how you look?""	Har du känt dig generad över hur du ser ut?<br>-> Har du känt dig blyg/osäker över hur du ser ut?
**Q2**	Did dealing with health problems upset you?	3(60)	The children do not understand the word "health problem" (n=2)<br><br>Instead of answering one of the response options, the child answers "no" (n=1)<br><br>The child says it is difficult to recall 4 weeks ago (n=1)		Yes	In Swedish health care, spina bifida is not automatically addressed as a disease, but a condition which requires the need for follow-up, monitoring and needing to take care of bladder/bowel function in order to stay healthy. This may contribute to the children's lack of understanding of "health problem". Additionally, "health problem" is an abstract term in Swedish language, which may impede the understanding by children who may have a cognitive dysfunction. Clarity of the item will be improved by adding relevant examples after the word "health problem." For example, "Did dealing with health problems upset you?" to "Were you upset (sad/angry) that you had to take care of your health (e.g. take enemas, use a catheter when peeing, use a corset or practice physiotherapy)?"	Har du blivit upprörd (ledsen/arg) av att ta hand om dina hälsoproblem?<br><br>-> Har du blivit upprörd (ledsen/arg) av att ta hand om din hälsa (ex ta lavemang, RIKa, sköta korsett eller sjukgymnastik)?
**Q3**	Did it bother you to catheterize to get urine out?	5(100)	Instead of answering one of the response options, the child answers "no" (n=1)		No		

Supplementary File S2.

**Q4**	Did it bother you if people in your family needed to help you?	5(100)	Instead of answering one of the response options, the child answers "no" (n=1)	Yes, I recognize this situation (n=2)	No		
**Q5**	Did it bother you if your friends or people outside your family needed to help you?	4(80)	I only receive help from my mother and father (n=1)		No		
**Bladder and Bowel**							
**Q6**	Did you worry someone would notice your pads, pull-ups or disposable underwear?	5(100)	Instead of answering one of the options, one child answers "no" (n=1)		No		
**Q7**	Did leaking urine bother you?	4(80)	Instead of answering one of the response options, the child answers "no" (n=1)<br><br>The child does not understand the question→ wishes to skip answering the question		No		
**Q8**	Did your urine problems stop you from doing fun things?	4(80)	The child does not understand the question→ wishes to skip answering the question	Understands the content in the question (n=1)	No		
**Q9**	Did leaking stool bother you?	4(100)	The children express a wish to skip answering the question (n=2)		No		
**Q10**	Did it bother you to wait for stool to come out?	5(100)			No		

* QUALAS is copyright-protected. Use can be licensed from the original instrument developer, Dr. Konrad Szymanski (szymanko@iu.edu)

Cognitive debriefing results of the Swedish version of questions included in QUALAS-T with teenagers (n=6) and decision-making process to change wording of Swedish translation

		**Teenagers' perception of the item as clear**	**Qualitative data**		**Project committee**		
**Questions (Q)***	**Original question in English**	**n(%)**	**Type difficulties encountered**	**Strength**	**Discussion about possible change**	**Discussion and consideration after the teenagers' input**	**Decision to change the wording of the Swedish translation**
**Family and Indepedence**							
**Q1**	Did it bother you if people in	6(100)		Recognizes and can relate to the situation (n=1)	No		

	your family needed to help you?						
**Q2**	Did it bother you if your friends or people outside your family needed to help you?	6(100)	The teenagers ask for clarification (n=2)	Can respond to the question using the response options (n=1)	No		
**Q3**	Did it annoy you if you could not do what other teenagers could do?	6(100)	The teenagers ask for clarification (n=2)	Recognizes and can relate to the situation (n=3)	No		
**Q4**	Did you worry about finding a boyfriend or girlfriend / husband or wife?	6(100)	Cannot relate to the situation (n=1)<br><br>The question was difficult to understand at first (n=1)<br><br>Needed clarification from the parent (n=2)<br><br>Difficult to use the response options (n=1)	Can relate to the question (n=1)	Yes	Five out of six teenagers expressed difficulties with understanding the Swedish translation in their comments. Based on the input from teenagers, the Swedish project committee suggested removing "husband and wife" as this was judged not applicable in a Swedish setting	Har du oroat dig över att kunna hitta en pojkvän eller flickvän / man eller fru?<br><br>-→ Har du oroat dig över att kunna hitta en pojkvän eller flickvän ~~/ man eller fru~~?
**Q5**	Did you worry about having children in the future , even if you already have a child?	6(100)	Cannot relate to the question (n=1)	Can relate to the question (n=1)	No		
**Bladder and Bowel**							

Supplementary File S2.

**Q6**	Did you worry someone would notice your pads, pull-ups or disposable underwear?	6(100)	The teenagers ask for clarification (n=1)<br><br>Difficult to use the response options (n=1)		No		
**Q7**	Did leaking urine bother you?	6(100)	The teenagers ask for clarification (n=1)<br><br>One teenager expresses a wish to skip answering the question (n=1)	Can relate to the question (n=2)<br><br>Can respond to the "not applicable" scale (n=1)	No		
**Q8**	Did your urine problems stop you from doing fun things?	6(100)	The teenagers ask for clarification (n=1)<br><br>Difficult to use the response options (n=1)		No		
**Q9**	Did leaking stool bother you?	6(100)	The question was difficult to understand at first (n=1)	Can relate to the question (n=1)<br>Can respond to the "not applicable" option (n=1)	No		
**Q10**	Did it bother you to wait for stool to come out?	6(100)	The teenagers ask for clarification (n=1)	Can relate to the question (n=1)<br><br>Can respond to the "not applicable scale (n=1)	No		

* QUALAS is copyright-protected. Use can be licensed from the original instrument developer, Dr. Konrad Szymanski (szymanko@iu.edu)

Cognitive debriefing results of the Swedish version of questions included in QUALAS-A with adults (n=5) and decision-making process to change wording of Swedish translation

		**Adults' perception of the item as clear**	**Qualitative data**		**Project committee**		
**Questions (Q)***	**Original question in English**	**n(%)**	**Type difficulties encountered**	**Strength**	**Discussion about possible change**	**Discussion and consideration after the adults' input**	**Decision to change the wording of the Swedish translation**
**Health and Relationship**							

Supplementary File S2.

**Q1**	Were you happy with your health?	4(80)		Recognizes and can relate to the situation (n=3)	No		
**Q2**	Did you feel people see you as a person and not only as a person with health problems?	5(100)	The adults ask for clarification (n=2)	Recognizes and can relate to the situation (n=2)	No		
**Q3**	Were you able to do fun things?	4(80)	The response options are difficult to use (n=1)	Recognizes and can relate to the situation (n=2)	No		
**Q4**	Did you spend time with friends your age outside school or work?	4(80)	The adult asks for clarification of the question (n=1)	Recognizes and can relate to the situation (n=3)	No		
**Q5**	Were you comfortable with your close friendships outside your family?	4(80)		Recognizes and can relate to the situation (n=3)	No		
**Esteem and Sexuality**							
**Q6**	Did you feel embarrassed about how you look?	3(60)	The response options are difficult to use (n=2)<br><br>The adult asks for clarification of the question (n=1)<br><br>The question was sensitive (n=1)		Yes		Har du känt dig generad över hur du ser ut?<br><br>-> Har du känt dig blyg/osäker över hur du ser ut?
**Q7**	Did it bother you if your family treated you differently because of your health problems?	5(100)	The adult asks for clarification (n=1) of the concept family (n=1)<br><br>The adult does not understand "bothered" (n=1)	Recognizes and can relate to the situation (n=1)	No		
**Q8**	Did you worry about having children in the future, even if	4(80)		Recognizes and can relate to the situation (n=3)	No		

Supplementary File S2.

	you already have a child?						
**Q9**	Were you happy with your sex life?	4(80)	The adult does not understand the question and asks for clarification (n=1)<br>The adult does not want to answer the question (n=1)	Recognizes and can relate to the situation (n=1)	No		
**Q10**	Did you think your health problems will affect how satisfied you will be with sexual activity in the future?	4(80)	Difficult to understand the relation between health problem and future activities (n=1)	Recognizes and can relate to the situation (n=2)	No		
**Bladder and Bowel**							
**Q11**	Did you worry someone would notice your pads, pull-ups or disposable underwear?	5(100)		Recognizes and can relate to the situation (n=1)	No		
**Q12**	Did leaking urine bother you?	5(100)			No		
**Q13**	Did your urine problems stop you from doing fun things?	4(80)		Recognizes and can relate to the situation (n=2)	No		
**Q14**	Did leaking stool bother you?	5(100)			No		
**Q15**	Did it bother you to wait for stool to come out?	5(100)			No		
* QUALAS is copyright-protected. Use can be licensed from the original instrument developer, Dr. Konrad Szymanski (szymanko@iu.edu)							
